# A Rare Case of Cutaneous Infection by *Corynascus verrucosus* in a Shih Tzu Dog: Molecular Identification and Phylogenetic Analysis

**DOI:** 10.1002/vms3.71205

**Published:** 2026-08-29

**Authors:** Mohammad Ghahremani, Aghil Sharifzadeh, Iradj Ashrafi Tamai, Ghazale Faridfar

**Affiliations:** ^1^ Department of Microbiology & Immunology Faculty of Veterinary Medicine University of Tehran Tehran Iran

**Keywords:** canine cutaneous lesion, *Corynascus verrucosus*, ITS rDNA, molecular diagnosis, sequencing, phylogeny, pruritus

## Abstract

**Background:**

Fungal infections are important causes of dermatological disease in companion animals and may involve a diverse range of clinically relevant fungal pathogens. Accurate identification of the causative agent is essential for appropriate diagnosis, treatment and assessment of potential zoonotic risks.

**Objectives:**

This study aimed to characterize a fungal isolate from a canine cutaneous lesion in Tehran, Iran, using morphological, molecular and phylogenetic analyses.

**Methods:**

Skin scrapings were aseptically collected from a dog presenting with pruritus and scaling, using sterile techniques microscopic examination and fungal culture. Microbiological observations were inconclusive for species‐level identification. Hence, target isolate was subjected to PCR amplification of the internal transcribed spacer (ITS) region using ITS1 and ITS4 universal primers.

**Results:**

The ITS1–5.8S rDNA–ITS2 region was successfully amplified and sequenced using the universal ITS1 and ITS4 primers, and the sequence was deposited in the NCBI GenBank (PV082530). BLAST analysis showed 99.36% nucleotide similarity to a reference sequence of *Corynascus verrucosus*. In the maximum likelihood analysis placed the Iranian isolate within the broader *Corynascus* lineage, clustering closely with *C. verrucosus* reference sequences from different geographic regions. Morphological findings, particularly large, round, verrucose conidia, supported the molecular identification. Overall, the combined morphological, molecular and phylogenetic findings supported identification as *C. verrucosus*.

**Conclusions:**

This case highlights the occurrence of an uncommon *C. verrucosus* isolate in a canine cutaneous lesion and demonstrates the value of integrating morphological, molecular, and phylogenetic approaches for fungal identification. The findings also emphasize the importance of cautious interpretation of molecular data when closely related fungal taxa show high sequence similarity.

## Graphical abstract

A 4‐year‐old Shih Tzu presented with severe pruritus, erythema, scaling and diffuse dorsal alopecia. A rare fungal isolate was recovered from the cutaneous lesion and showed characteristic colonies and verrucose conidia. ITS1–5.8S–ITS2 sequencing (GenBank PV082530) showed similarity to *Corynascus verrucosus*. Phylogenetic analysis supported its placement within the *Corynascus* lineage. Integrated morphological, molecular and phylogenetic evidence supported identification as *C. verrucosus*. This case highlights the value of integrated approaches for identifying uncommon fungal isolates in veterinary dermatology.

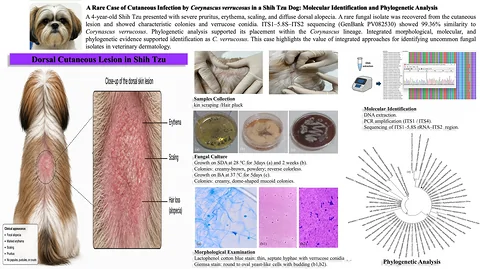

## 1 Introduction

Fungal infections commonly play a significant role in dermatological conditions affecting companion animals. Dermatophytosis in small animals is a cutaneous disorder resulting from superficial fungal infections of keratinized skin tissues. The most frequently implicated agents are zoophilic, geophilic or anthropophilic fungi, particularly *Microsporum canis*, *Microsporum gypseum* and *Trichophyton mentagrophytes*. Due to its variable clinical presentation, contagious nature and zoonotic potential, dermatophytosis represents an important concern in small animal veterinary medicine. Although no single diagnostic test has been established as a gold standard, diagnosis is generally based on a combination of complementary methods, including Wood's lamp examination, direct microscopy and fungal culture. The review also indicated that effective treatment generally involves systemic antifungal therapy combined with topical hair‐coat disinfection. Wood's lamp examination and direct microscopy may provide useful diagnostic information, while environmental decontamination through thorough physical cleaning remains an important component of disease control (Mueller et al. 2020).

Although dermatophytes are the predominant causative pathogens of fungal skin infections, infections caused by environmental or saprophytic fungi are less commonly reported and may remain undiagnosed. *Corynascus verrucosus* is an environmental fungus reported from soil and plant‐associated environments (Sharma et al. 2013), and its recovery from a canine cutaneous lesion in the present case is noteworthy. The genus *Corynascus* has been described as comprising seven recognized species: *Corynascus heterothallicus*, *Corynascus novoguineensis*, *Corynascus sepedonium*, *Corynascus sexualis*, *Corynascus similis*, *Corynascus thermophilus* and *C. verrucosus* (van den Brink et al. 2012). The taxonomy of *Corynascus* has been developed through a series of taxonomic studies, including those by von Klopotek (1974) and Stchigel et al. (2000). Stchigel et al. (2000) further contributed to the taxonomy of *Corynascus* by describing three new thermotolerant species and providing a key to the known species. Marin‐Felix et al. (2015) re‐evaluated the taxonomy of *Myceliophthora* using morphological and multilocus phylogenetic data, resulting in the recognition of *Corynascus* as a distinct genus. Mohana et al. (2020) provided the first documented evidence of *C. verrucosus* as a mycoendophyte associated with *Croton bonplandianus* Baill. and characterized its bioactive potential, highlighting this fungus as a potential source of natural products with activity against multidrug‐resistant pathogens and cancer.

Recent studies have demonstrated substantial changes in fungal and bacterial community composition and diversity throughout different stages of organic waste and manure composting, with microbial succession closely associated with changes in physicochemical conditions (Zhao et al. 2023; Duan et al. 2019; Galitskaya et al. 2017). Composting environments can support diverse thermophilic and thermotolerant fungi, including taxa adapted to elevated temperatures and changing substrate conditions (Duan et al. 2019; Galitskaya et al. 2017). Subsequent studies have documented diverse thermophilic and thermotolerant fungal communities in composting environments, highlighting these ecosystems as important reservoirs of environmentally adapted fungal taxa (K. Wang et al. 2018; Langarica‐Fuentes et al. 2014). These findings suggest that composting and other organic‐material environments may serve as potential reservoirs of environmentally adapted fungi.

In parallel, phylogenetic analyses based on ITS‐rDNA and other genetic markers have been used to investigate the relationships among thermophilic *Corynascus* species and related *Myceliophthora* taxa, supporting their close phylogenetic relationship and contributing to the refinement of their taxonomic placement (van den Brink et al. 2012; Marin‐Felix et al. 2015; Huang and Jiang 2015). Among these environmental fungi, species within *Corynascus* and the closely related genus *Myceliophthora* share substantial phylogenetic similarity. Members of the *Myceliophthora* lineage have attracted considerable interest for their industrial and biotechnological applications, including the production of thermostable enzymes (Zanphorlin et al. 2010). However, phylogenetic relatedness does not necessarily imply similar biological functions or pathogenic potential, and genetic similarity should therefore not be interpreted as evidence of a shared pathogenic role. Precise identification of fungal species, particularly those isolated from environmental samples, can also be challenging due to morphological similarities among closely related taxa. Nevertheless, molecular techniques targeting ribosomal DNA regions, particularly the ITS1–5.8S–ITS2 region, provide valuable complementary information for fungal identification and phylogenetic analysis (White et al. 1990).

Accordingly, the recovery of an uncommon environmental fungus from a canine cutaneous lesion underscores the importance of integrating phenotypic, molecular, and phylogenetic evidence for accurate fungal identification in veterinary clinical specimens, particularly for fungal isolates with limited or inconclusive morphological characteristics, while recognizing that phylogenetic relatedness alone does not establish pathogenicity.

## 2 Case Presentation

A 4‐year‐old, spayed female Shih Tzu was referred to the Veterinary Clinic of the Faculty of Veterinary Medicine, University of Tehran, Iran. The dog was housed outdoors under unsanitary conditions and had regular exposure to vegetation and soil. The patient presented with diffuse alopecia, marked erythema, pruritus and scaling involving the dorsal skin, without ulceration or apparent wound formation. The animal showed no signs of lethargy or anorexia. Because no systemic clinical signs were observed, haematological examination was not performed. Hair and skin scrapings were subsequently collected from the affected areas under sterile conditions and submitted to the laboratory for fungal examination. Histopathological examination was not performed because the owner did not provide consent for skin biopsy. Therefore, direct demonstration of fungal elements within the affected tissue was not possible.

**FIGURE 1:**
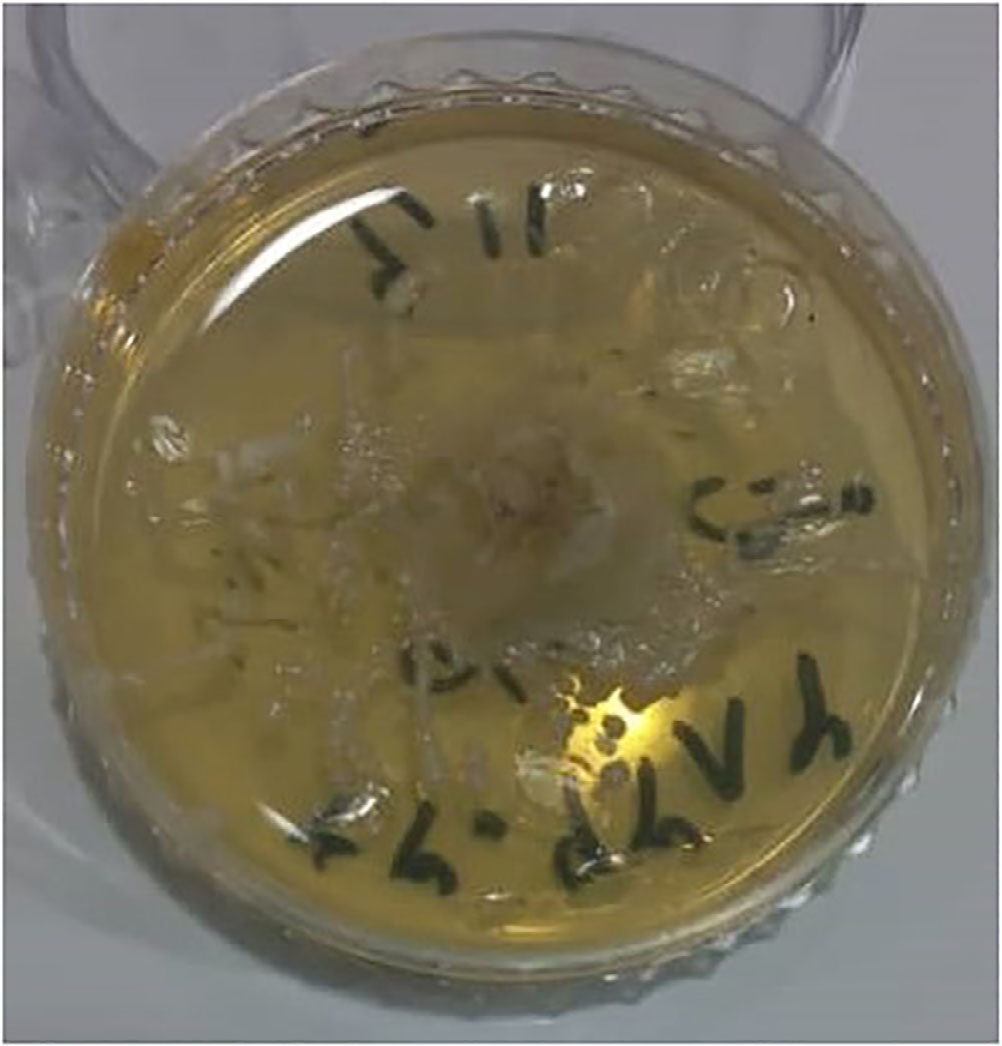
Colony morphology on Sabouraud Dextrose Agar at 28°C for 3 days.

**FIGURE 2:**
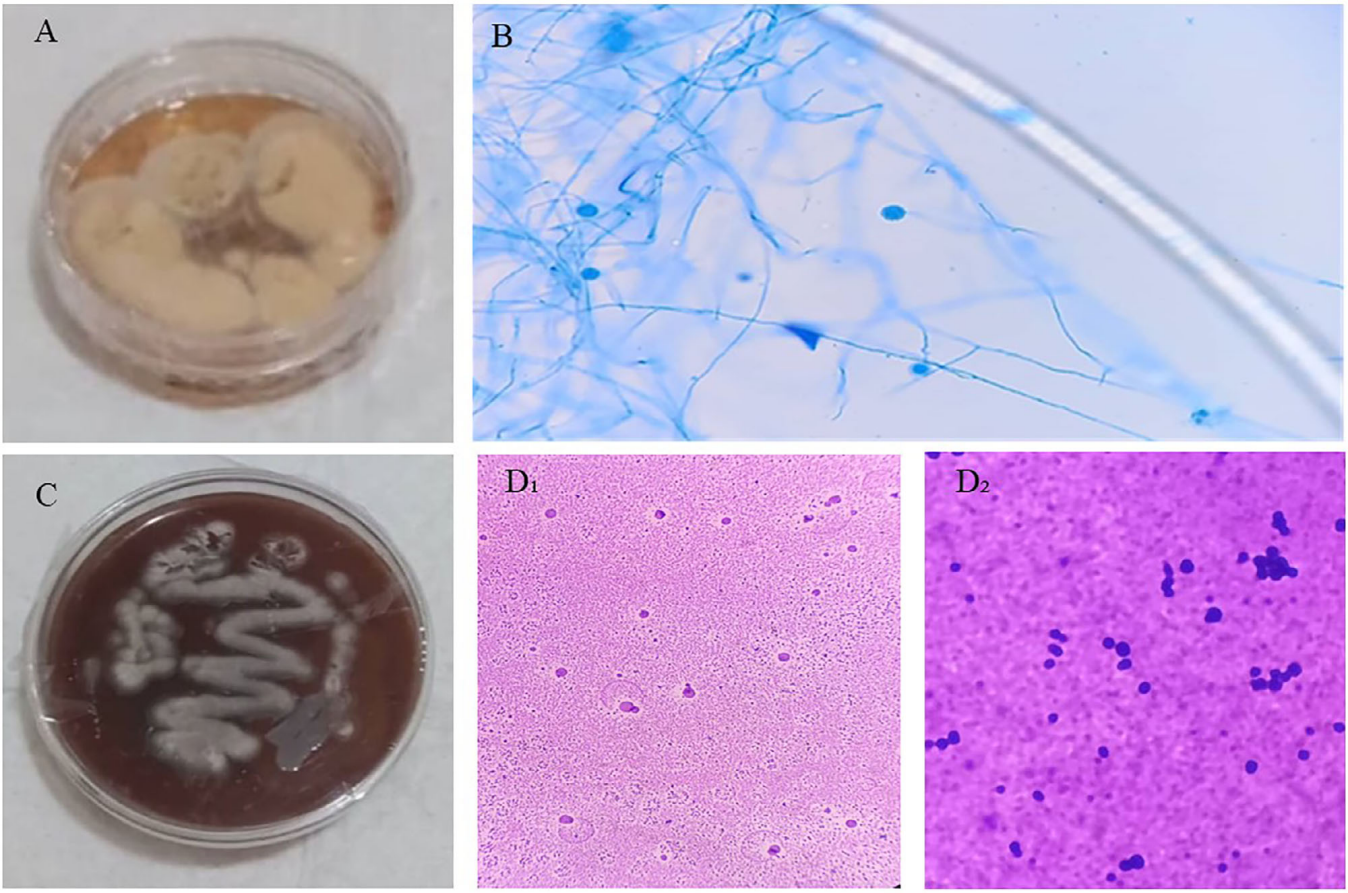
Colony morphology (A) and conidia (B) on Sabouraud Dextrose Agar (SDA), incubated at 28°C for 2 weeks. Colony morphology (C) and yeast‐like cells (D1, D2) on blood agar, incubated at 37°C for 5 days (100× magnification).

In clinical examination, Wood's lamp test was negative. Samples of hair and skin scrapings were taken from the affected regions under sterile conditions and sent to the lab for examination. For direct microbiologic analysis, 10% KOH examination was negative. Fungal culture was performed by inoculating the sample onto Sabouraud Dextrose Agar (SDA) and incubating it at 28°C for 3 days (Figure 1) and 2 weeks (Figure 2A), respectively. Colonies appeared creamy‐brown and powdery, with a colourless reverse. Diagnostic testing ruled out dermatophyte involvement. Further, lactophenol cotton blue staining revealed thin, septate hyphae with large, round verrucose conidia (Figure 2B). Next, colonies were subcultured onto blood agar and incubated at 37°C for 5 days, resulting in creamy, dome‐shaped mucoid colonies (Figure 2C). Giemsa staining also revealed further round‐to‐oval yeast‐like cells with budding and point attachments under oil immersion lens 100× (Figure 2D1,2).

**FIGURE 3:**
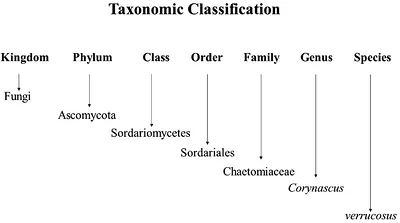
Taxonomic classification of *Corynascus verrucosus* within the family Chaetomiaceae.

Due to inconclusive morphological findings, the target isolate was subjected to PCR amplification of the internal transcribed spacer (ITS) region using ITS1 and ITS4 universal primers (Table 1). Molecular identification was performed using a fungal DNA extraction kit (QIAGEN) to extract genomic DNA from the isolate. The ITS region, comprising ITS1, the 5.8S rDNA gene and ITS2, was amplified using the universal ITS1 and ITS4 primers for species‐level identification (White et al. 1990). The PCR conditions were as follows: initial denaturation at 94°C for 3 min; 37 cycles of denaturation at 94°C for 30 s, annealing at 60°C for 30 s, and extension at 72°C for 30 s; followed by a final extension at 72°C for 8 min. The primer sequences (5′–3′) are presented in Table 1. The amplified ITS region was sequenced, and the resulting sequence was deposited in GenBank under accession number PV082530. The sequence was aligned with reference sequences retrieved from GenBank. Phylogenetic analysis was performed using MEGA7 based on the maximum likelihood method, with the Kimura 2‐parameter model and 1000 bootstrap replicates. The Iranian isolate was placed within the broader *Corynascus* lineage and clustered closely with reference sequences of *C. verrucosus*. The phylogenetic findings were consistent with previously published taxonomic data (Figure 3), supporting the placement of the isolate within the family Chaetomiaceae (Sastoque et al. 2025; Q. Wang et al. 2023).

**TABLE 1:** Sequence and characteristics of primers used for amplification of the ITS region in *C. verrucosus*.

Gene	Primers	Sequence (5’‐3’)	Annealing Temperature (°C)	Length	Size (bp)
ITS1	Forward	TCC GTA GGT GAA CCT GCG G	61.64	19	**∼**600
ITS4	Revers	TCC TCC GCT TAT TGA TAT GC	55.09	20	

**FIGURE 4:**
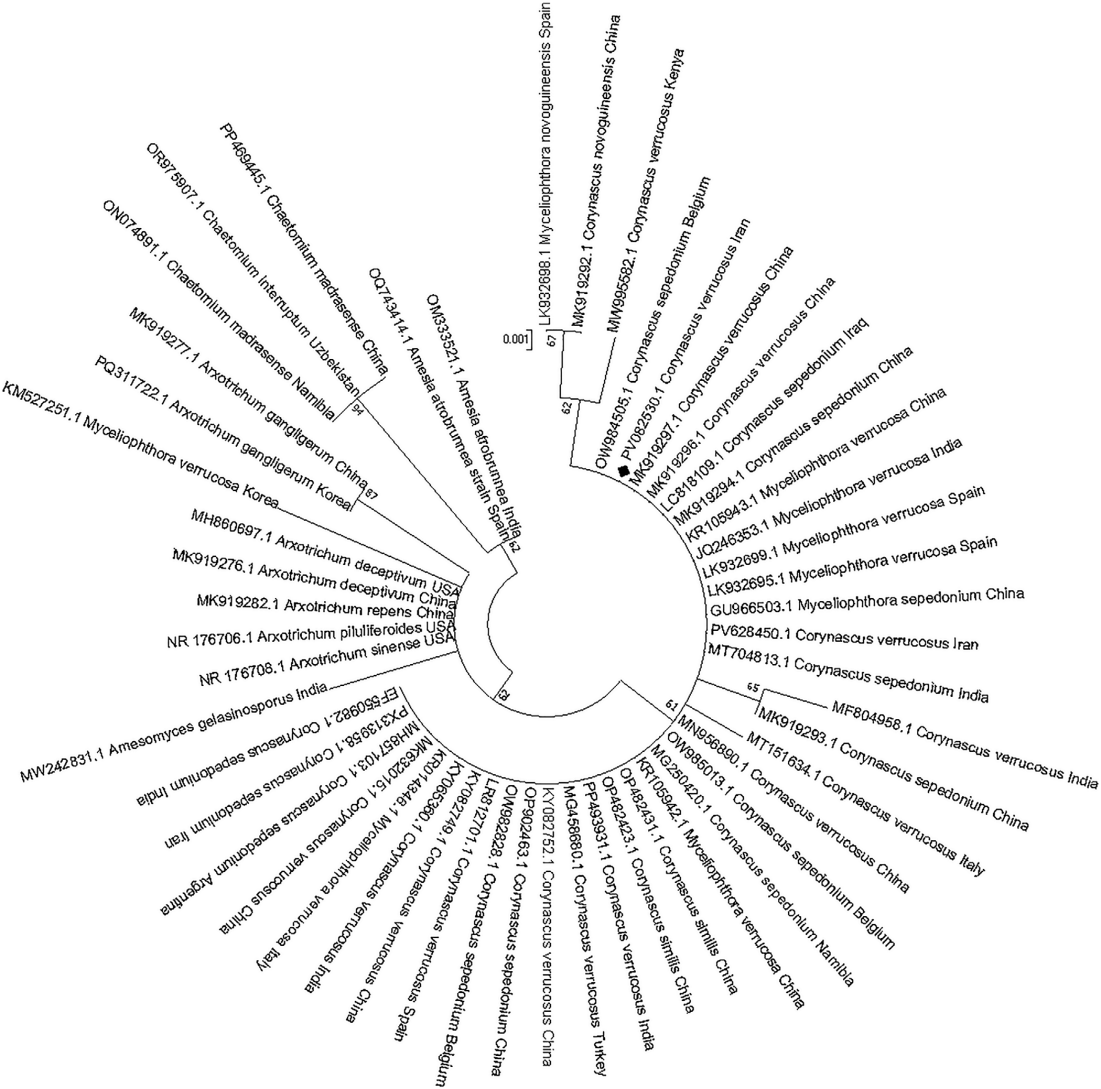
Maximum likelihood phylogenetic analysis based on ITS1–5.8S rDNA–ITS2 sequences of *Corynascus* and related taxa. The phylogenetic tree was constructed using the maximum likelihood method with 1000 bootstrap replicates. The codes preceding the taxon names represent GenBank accession numbers. Numbers at the nodes indicate bootstrap support values, and the scale bar represents 0.001 nucleotide substitutions per site. The tree was rooted using *Amesia atrobrunnea* (OM333521.1 and OQ743414) as outgroup taxa.

Sequence analysis of the 5.8S rDNA gene and flanking ITS regions serves as a highly informative molecular marker as well as providing robust phylogenetic resolution for fungal identification. It likewise highlights the importance of accurate fungal identification in atypical dermatological manifestations. Although morphological identification was limited, molecular data confirmed the identity of the isolate as *C. verrucosus* (Figure 4). This case can therefore reinforce the utility of molecular approaches in environmental mycology, especially for cryptic fungal species. The repeated isolation of the same fungal species from the affected skin lesion on three independent cultures, together with the absence of bacterial growth on culture, supports its clinical association with the lesion. However, in the absence of histopathological evidence of tissue invasion, a definitive causal role cannot be established. This report expands the available knowledge on the occurrence of *C. verrucosus* in a canine cutaneous lesion. In the present study, ITS sequencing was therefore interpreted together with morphological characteristics and phylogenetic placement rather than being considered as the sole criterion for species identification.

The dog was treated with oral ketoconazole in combination with ketoconazole shampoo, with four weekly baths administered. Pruritus and erythema resolved within approximately 1 month, followed by gradual hair regrowth. A uniform hair coat was observed approximately 6 months after treatment, and no recurrence of the initial cutaneous signs was observed during the 9‐month follow‐up period. The clinical improvement following antifungal therapy, together with repeated recovery of the same fungal isolate from the lesion and its morphological and molecular identification, supports a clinical association between *C. verrucosus* and the cutaneous lesion.

## 3 Discussion

*C. verrucosus* is primarily recognized as an environmental fungus and has not been well established as a pathogenic organism in veterinary or human medicine. Therefore, its recovery from a canine cutaneous lesion should be interpreted cautiously, and its potential role as an opportunistic organism cannot be distinguished from environmental colonization or contamination based solely on the available evidence. To the best of our knowledge, this case represents a rare report of the recovery of *C. verrucosus* from a canine cutaneous lesion. The isolate was recovered repeatedly in culture from hair and skin scale samples collected from the affected lesion, while no bacterial growth was detected. The ITS1–5.8S rDNA–ITS2 region was successfully amplified and sequenced, and the resulting sequence was deposited in GenBank under accession number PV082530. BLAST analysis showed 99.36% nucleotide similarity to reference sequences of *C. verrucosus* and 99.34% similarity to a reference sequence of *C. sepedonium*. In the maximum likelihood phylogenetic analysis, the isolate clustered closely with reference sequences assigned to *C. verrucosus* from different geographic regions and was positioned within the broader *Corynascus* lineage. Reference sequences annotated as *Myceliophthora*, including *Myceliophthora sepedonium* and *Myceliophthora verrucosa*, were also positioned within the same broader phylogenetic assemblage, reflecting the close taxonomic relationship among these taxa. The molecular findings were consistent with the morphological characteristics of the isolate, particularly the presence of large, round, verrucose conidia, a characteristic feature reported for *C. verrucosus* (Sharma et al. 2013). However, given the high ITS similarity with closely related taxa, including *C. sepedonium*, the phylogenetic results should be interpreted as supportive evidence for taxonomic identification rather than independent proof of pathogenicity.

Our phylogenetic findings were consistent with previously published taxonomic and phylogenetic data, supporting the placement of the isolate within the genus *Corynascus* and family Chaetomiaceae. The ITS‐rDNA phylogenetic analysis revealed a close relationship among *C. verrucosus*, *C. sepedonium*, and *M. verrucosa*, with these taxa occurring within a closely related phylogenetic assemblage, highlighting the limited discriminatory power of the ITS region among closely related taxa. The close clustering of *Corynascus* and *Myceliophthora* sequences in the present ITS phylogeny is consistent with previous phylogenetic studies reporting a close relationship between these taxa (van den Brink et al. 2012). However, recent comprehensive studies have supported the distinction of *Corynascus* from *Myceliophthora* based on multigene phylogenetic analyses combined with morphological characteristics (X. W. Wang et al. 2022). Therefore, the present identification was interpreted by integrating morphological characteristics, ITS sequence similarity and phylogenetic placement rather than relying solely on ITS sequence similarity. This integrated approach provides stronger support for the taxonomic identification of uncommon environmental fungal isolates and may help reduce the risk of misidentification or incomplete classification.

On the other hand, morphological identification of Chaetomiaceae may be challenging because closely related taxa can exhibit similar phenotypic characteristics. Therefore, molecular and phylogenetic analyses can provide supportingevidence for species‐level identification (Al‐Rifaie and Ameen 2023). Molecular identification can also be particularly useful for fungal isolates that fail to produce characteristic reproductive or sporulating structures in culture, where morphological identification may be limited (Huang and Jiang 2015). However, molecular approaches may also be influenced by methodological biases and should therefore be interpreted in conjunction with morphological and clinical findings (Shinohara et al. 2021). Accordingly, Huang and Jiang (2015) successfully used ITS‐rDNA sequences to support the identification of *C. verrucosus* among endophytic fungi, while emphasizing the value of integrating molecular and morphological characteristics for fungal identification. Taken together, recent taxonomic studies of Chaetomiaceae have emphasized a polyphasic approach combining morphological characterization with multilocus phylogenetic analyses, including ITS, LSU, rpb2, tub2 and tef, for reliable taxonomic delimitation (Sastoque et al. 2025).

This case highlights the value of integrating morphological, molecular and phylogenetic approaches for the identification of uncommon fungal isolates from veterinary clinical specimens. From a clinical perspective, the repeated recovery of the same fungal isolate from the affected skin lesion further supports its clinical association with the case. Although histopathological confirmation of fungal invasion was not available, repeated isolation of the same fungal species from the lesion, exclusion of bacterial infection and molecular identification suggest that *C. verrucosus* may have contributed to the observed cutaneous condition. Thus, this case expands the available knowledge on the occurrence of *C. verrucosus* in a canine cutaneous lesion and highlights the need for cautious interpretation of uncommon environmental fungal isolates in veterinary clinical specimens.

## 4 Limitation

A limitation of the present case report was the absence of histopathological examination because the owner did not consent to skin biopsy. Consequently, fungal invasion of the tissue could not be directly demonstrated, and the clinical role of the isolate should therefore be interpreted as an association rather than definitive evidence of causality. Although repeated isolation of the same fungal isolate from the affected skin lesion, together with the absence of bacterial growth and the exclusion of mite infestation, supports its clinical association with the lesion. Nevertheless, definitive causality cannot be established in the absence of histopathological evidence. This report expands the available knowledge on the occurrence of *C. verrucosus* in a canine cutaneous lesion.

## Author Contributions

**Mohammad Ghahremani**: conceptualization, data curation, visualization. **Aghil Sharifzadeh**: conceptualization, formal analysis, supervision, validation, methodology. **Iradj Ashrafi Tamai**: conceptualization, formal analysis, validation. **Ghazale Faridfar**: conceptualization, methodology, software, writing – original draft, writing – review and editing. All authors contributed to the article and approved the submitted version.

## Funding

The authors have nothing to report.

## Ethics Statement

The authors have nothing to report.

## Conflicts of Interest

The authors declare no conflicts of interest.

## Supporting information



Supporting File 1: vms371205‐sup‐0001‐SuppMat.docx

## Data Availability

The raw datasets used in this study are available from the authors upon reasonable request, without undue reservation.

## References

[vms371205-bib-0001] Al‐Rifaie, A. A. , and M. K. M. Ameen . 2023. “New Endophytic Fungal Species of Chaetomiaceae (Ascomycota) in Iraq.” Biodiversitas 24, no. 10: 5270–5277. 10.13057/biodiv/d241007.

[vms371205-bib-0002] Duan, Y. , S. K. Awasthi , T. Liu , et al. 2019. “Dynamics of Fungal Diversity and Interactions With Environmental Elements in Response to Wheat Straw Biochar Amended Poultry Manure Composting.” Bioresource Technology 274: 410–417. 30551044 10.1016/j.biortech.2018.12.020

[vms371205-bib-0003] Galitskaya, P. , L. Biktasheva , A. Saveliev , T. Grigoryeva , E. Boulygina , and S. Selivanovskaya . 2017. “Fungal and Bacterial Successions in the Process of Co‐Composting of Organic Wastes as Revealed by 454 Pyrosequencing.” PLoS ONE 12, no. 10: e0186051. 10.1371/journal.pone.0186051. 29059245 PMC5653195

[vms371205-bib-0004] Huang, J. , and Z. Jiang . 2015. “Phylogenetic Analysis of Endophytes From Bitter Melon (*Momordica charantia*) in Guangdong Province.” Agricultural Sciences 6, no. 6: 609–621. 10.4236/as.2015.66060.

[vms371205-bib-0005] Langarica‐Fuentes, A. , P. S. Handley , A. Houlden , G. Fox , and G. D. Robson . 2014. “An Investigation of the Biodiversity of Thermophilic and Thermotolerant Fungal Species in Composts Using Culture‐Based and Molecular Techniques.” Fungal Ecology 11: 132–144. 10.1016/j.funeco.2014.05.007.

[vms371205-bib-0006] Marin‐Felix, Y. , A. M. Stchigel , A. N. Miller , J. Guarro , and J. F. Cano‐Lira . 2015. “A Re‐Evaluation of the Genus *Myceliophthora* (Sordariales, Ascomycota): Its Segregation Into Four Genera and Description of *Corynascus fumimontanus* sp. nov.” Mycologia 107, no. 3: 619–632. 10.3852/14-228. 25661719

[vms371205-bib-0007] Mohana, N. C. , D. Rakshith , H. Y. Rao , K. P. Ramesha , B. R. Nuthan , and S. Satish . 2020. “Bioassay Guided Fractionation of Bioactive Metabolite From *Corynascus verrucosus* Inhabiting *Croton bonplandianus* Baill.” Process Biochemistry 98: 106–112. 10.1016/j.procbio.2020.07.007.

[vms371205-bib-0008] Mueller, R. S. , W. Rosenkrantz , E. Bensignor , J. Karaś‐Tęcza , T. Paterson , and M. A. Shipstone . 2020. “Diagnosis and Treatment of Demodicosis in Dogs and Cats: Clinical Consensus Guidelines of the World Association for Veterinary Dermatology.” Veterinary Dermatology 31, no. 1: 5–27. 10.1111/vde.12806. 31957202

[vms371205-bib-0009] Sastoque, A. P. , J. F. Cano‐Lira , and A. M. Stchigel . 2025. “Soil Ascomycetes From Spain. XIV. The Chaetomiaceae of La Palma (Canary Islands).” Persoonia 54, no. 1: 93–117. 10.3114/persoonia.2025.54.03. 40746711 PMC12308285

[vms371205-bib-0010] Sharma, R. , G. Kulkarni , and Y. S. Shouche . 2013. “ *Corynascus verrucosus*, Second Record for Science and New for India.” Nova Hedwigia 97, no. 3–4: 485–494. 10.1127/0029-5035/2013/0112.

[vms371205-bib-0011] Shinohara, N. , C. Woo , N. Yamamoto , K. Hashimoto , H. Yoshida‐Ohuchi , and Y. Kawakami . 2021. “Comparison of DNA Sequencing and Morphological Identification Techniques to Characterize Environmental Fungal Communities.” Scientific Reports 11: 2633. 10.1038/s41598-021-81996-w. 33514828 PMC7846767

[vms371205-bib-0012] Stchigel, A. M. , M. Sagues , J. Cano , and J. Guarro . 2000. “Three New Thermotolerant Species of *Corynascus* From Soil, With a Key to the Known Species.” Mycological Research 104: 879–887.

[vms371205-bib-0013] van den Brink, J. , R. A. Samson , F. Hagen , T. Boekhout , and R. P. de Vries . 2012. “Phylogeny of the Industrial Relevant, Thermophilic Genera *Myceliophthora* and *Corynascus* .” Fungal Divers 52: 197–207.

[vms371205-bib-0014] von Klopotek, A. 1974. “Revision of Thermophilic *sporotrichum* Species*: Chrysosporium thermophilum* (Apinis) Comb nov. and Chrysosporium fergusii spec. nov., Equal Status Conidialis of Corynascus thermophilus Fergus and (Sinden) Comb. nov.” Archives of Microbiology 98: 365–369. 4858767 10.1007/BF00425296

[vms371205-bib-0015] Wang, K. , X. B. Yin , H. L. Mao , C. Chu , and Y. Tian . 2018. “Changes in Structure and Function of Fungal Community in Cow Manure Composting.” Bioresource Technology 255: 123–130. 29414157 10.1016/j.biortech.2018.01.064

[vms371205-bib-0016] Wang, Q. , L. Wang , L. Lian , et al. 2023. “Case Report: A Case of Ocular Infection Caused by *Corynespora cassiicola* .” Frontiers in Cellular and Infection Microbiology 13: 1160831. 10.3389/fcimb.2023.1160831. 37448776 PMC10338080

[vms371205-bib-0017] Wang, X. W. , P. J. Han , F. Y. Bai , et al. 2022. “Taxonomy, Phylogeny and Identification of Chaetomiaceae With Emphasis on Thermophilic Species.” Studies in Mycology 101: 121. 10.3114/sim.2022.101.03. 36059895 PMC9365047

[vms371205-bib-0018] White, T. J. , T. Bruns , S. Lee , and J. Taylor . 1990. “Amplification and Direct Sequencing of Fungal Ribosomal RNA Genes for Phylogenetics.” In PCR Protocols: A Guide to Methods and Applications, edited by M. A. Innis , D. H. Gelfand , J. J. Sninsky , and T. J. White , 315–322. Academic Press.

[vms371205-bib-0019] Zanphorlin, L. M. A. , F. D. A. Facchini , F. Vasconcelos , et al. 2010. “Production, Partial Characterization, and Immobilization in Alginate Beads of an Alkaline Protease From a New Thermophilic Fungus *Myceliophthora* sp.” Journal of Microbiology 48: 331–336. 10.1007/s12275-010-9269-8. 20571951

[vms371205-bib-0020] Zhao, X. , J. Li , H. Yuan , Z. Che , and L. Xue . 2023. “Dynamics of Bacterial Diversity and Functions With Physicochemical Properties in Different Phases of Pig Manure Composting.” Biology 12, no. 9: 1197. 10.3390/biology12091197. 37759597 PMC10525911

