# Correction to “A Rare Case of Cutaneous Infection by *Corynascus verrucosus* in a Shih Tzu Dog: Molecular Identification and Phylogenetic Analysis"

**DOI:** 10.1002/vms3.71296

**Published:** 2026-10-21

Ghahremani, M., A. Sharifzadeh, I. Ashrafi Tamai, and G. Faridfar. 2026. “A Rare Case of Cutaneous Infection by *Corynascus verrucosus* in a Shih Tzu Dog: Molecular Identification and Phylogenetic Analysis.” *Veterinary Medicine and Science* 12, no. 5: e71205. https://doi.org/10.1002/vms3.71205.

An additional email address has been added for the corresponding author, Aghil Sharifzadeh. The updated Correspondence section is as follows:

“Correspondence: Aghil Sharifzadeh (asharifzadeh@ut.ac.ir; asharifzadeh1363@gmail.com)”

The online version of this article has been corrected accordingly.

We apologize for this error.

