# Supplementary material for: A Rare Case of Cutaneous Infection by Corynascus verrucosus in a Shih Tzu Dog: Molecular Identification and Phylogenetic Analysis
Source: Vet Med Sci. 2026 Aug 29;12(5):e71205. doi: 10.1002/vms3.71205 (PMC13525849; doi:10.1002/vms3.71205)
Supplement: Supplementary file 1 [file VMS3-12-e71205-s001.docx]

A 4-year-old Shih Tzu presented with severe pruritus, erythema, scaling, and diffuse dorsal alopecia. A rare fungal isolate was recovered from the cutaneous lesion and showed characteristic colonies and verrucose conidia. ITS1–5.8S–ITS2 sequencing (GenBank PV082530) showed 99.36% similarity to *Corynascus verrucosus*. Phylogenetic analysis supported its placement within the *Corynascus* lineage. Integrated morphological, molecular, and phylogenetic evidence supported identification as *C. verrucosus*. This case highlights the value of integrated approaches for identifying uncommon fungal isolates in veterinary dermatology.
